# Gene-Specific Sex Effects on Susceptibility to Infectious Diseases

**DOI:** 10.3389/fimmu.2021.712688

**Published:** 2021-10-14

**Authors:** Marie Lipoldová, Peter Demant

**Affiliations:** ^1^ Laboratory of Molecular and Cellular Immunology, Institute of Molecular Genetics of the Czech Academy of Sciences, Prague, Czechia; ^2^ Department of Molecular and Cellular Biology, Roswell Park Comprehensive Cancer Center, Buffalo, NY, United States

**Keywords:** sex-bias, sex-dependent gene, mouse model, susceptibility to infection, sex influence, viruses, bacteria, parasites

## Abstract

Inflammation is an integral part of defense against most infectious diseases. These pathogen-induced immune responses are in very many instances strongly influenced by host’s sex. As a consequence, sexual dimorphisms were observed in susceptibility to many infectious diseases. They are pathogen dose-dependent, and their outcomes depend on pathogen and even on its species or subspecies. Sex may differentially affect pathology of various organs and its influence is modified by interaction of host’s hormonal status and genotype: sex chromosomes X and Y, as well as autosomal genes. In this Mini Review we summarize the major influences of sex in human infections and subsequently focus on 22 autosomal genes/loci that modify in a sex-dependent way the response to infectious diseases in mouse models. These genes have been observed to influence susceptibility to viruses, bacteria, parasites, fungi and worms. Some sex-dependent genes/loci affect susceptibility only in females or only in males, affect both sexes, but have stronger effect in one sex; still other genes were shown to affect the disease in both sexes, but with opposite direction of effect in females and males. The understanding of mechanisms of sex-dependent differences in the course of infectious diseases may be relevant for their personalized management.

## Introduction

Sex plays an important role in immune response, including susceptibility to infectious diseases (1), outcome of vaccination (2–6) and response to treatment (7). Sex differences in susceptibility to infectious and inflammatory diseases are widespread – both in terms of number of pathogens and diseases they influence and in terms of the number of vertebrate and invertebrate species and genera where they were observed. In humans they were demonstrated in a number of diseases discussed in detail below and hence they form a significant but hitherto unexplained component of clinical inter-patient heterogeneity. Their individual prediction and functional explanation may therefore significantly improve individual management of disease. A part of this phenomenon may be under genetic control, but there is presently little evidence for this in humans. However, there are extensive data from studies in mice that described 22 autosomal gene-loci controlling the sex differences in response to 12 infectious or inflammatory agents. We are presenting a comprehensive summary of this information, as it may help to proceed to clarification of the manifestations and mechanisms of sex differences in these pathologies.

Sexual dimorphism takes place already in healthy individuals. In most cases, basal immune responses are higher in females than in males. It has been described that women have higher several immunology-related parameters than males: blood levels of mature B cell subsets, IgM-only B cells, proliferating and memory (CD45RA-) Treg cells, NK bright (CD56++CD16-) subsets (8), immunoglobulin M (9), neutrophil and platelets (10), and higher CD4+/CD8+ ratio (11). TLR7 ligands induce higher IFNα production in woman peripheral blood lymphocytes (12). Neutrophils of men exhibit lower responses to cytokine stimulation and decreased ability to form neutrophil traps (13), whereas in women neutrophils were characterized by enhanced type I IFN pathway activity and enhanced proinflammatory responses (14). Male and female neutrophils differ also in bioenergetics. Metabolic assays of oxygen consumption rate (OCR), which is a key metric of mitochondrial function, and the extracellular acidification rate (ECAR), which approximates glycolytic activity in male and female neutrophils shown that OCR was higher in male than female neutrophils, whereas there were no differences in ECAR (14). As the immune cells differentiation and function crucially depend on mitochondrial bioenergetics (15, 16), sex differences in mitochondrial functions have a potential to modulate immune responses. Differences in immune responses have been observed also between males and females of other mammals (17–19), birds (20), reptiles (21), echinoderms (22) and insects (23). Such baseline differences can contribute to sex biases in response to pathogens (24,25 and see the next section) and to vaccination (2–6). Females usually have more efficient response to vaccination than males (2–5), but also develop more often adverse reactions to vaccination (4, 5). On the other hand, vaccination of healthy volunteers by Bacille Calmette-Guérin (BCG) led to enhanced cytokine responses to restimulation and reduced systemic inflammation. The effect was much stronger in men than in women (6).

## Sex Biases in Human Infections

### Viruses

Male sex was associated with higher death rate in hepatitis A virus-hospitalized cases (24). Male sex was also a risk factor for hepatitis B virus (HBV) and hepatitis C Virus (HCV) prevalence and for development of hepatocellular carcinoma subsequent to HBV and/or HCV infection (25). Presence of virus stimulates inflammatory responses and appearance of reactive oxygen (ROS) and nitrogen species, which are described as leading cause of series of alterations that led to DNA damage (25). Estrogen may serve an inhibitory role in these processes by inhibiting inflammation, tumor progression and invasion and stimulating DNA repair (26), whereas androgen induced miR-216a stimulated tumorigenesis (27). The lower survival rate was observed among male patients infected with Ebola virus (28). The higher COVID-19 case mortality rate and increased severity of disease was described in males (29–31). Interestingly, gene encoding ACE2 (angiotensin-converting enzyme 2), which plays an essential role in cell entry of SARS-CoV-2 (severe acute respiratory syndrome coronavirus 2) is localized on X chromosome (Xp22.2), thus females have double gene dose and can be potentially heterozygous compared to males who are definitely hemizygous. It has been speculated that together with X mosaicism it might favor women in counteracting the progression of the SARS-CoV-2 infection (32, 33). Prevalence of herpes simplex virus type 1 (HSV1) and type 2 (HSV2) in persons aged 14-49 in United States in the years 2015-2016 was higher in women than in men (34). Similarly, HSV1 in Europe was more often detected in women than in men (35). Sex differences in measles mortality were compared among 78 countries in years between 1950 and 1989. Regional variations showed excess female mortality of 3% in Europe, 6.2% in North America, 5.9% in Far East Asia, 4.3% in Latin America, and 20.9% in the Middle East. The cumulative excess female mortality in comparison with males was small at age 0-4 (+4.2%), larger at age 5-14 (+10.9%), and peaks at ages 15-44 (+42.6%) (36). The most probable explanation of these variations is the influence of estrogens (36). Several studies have shown that women are more susceptible to human immunodeficiency virus 1 (HIV-1) acquisition than men, as the male-to-female transmission is more efficient than female-to-male transmission (37, 38). Indeed, in Sub-Saharan Africa higher HIV prevalence is observed in women (39). However, in Europe, there are more newly detected HIV infections in males than in woman, because sex between men remains the predominant mode of HIV transmission reported in the EU (European Union)/EEA (European Economic Area) (40). Thus, socioeconomic factors most likely contribute to the sex biases in HIV/AIDS (Acquired Immune Deficiency Syndrome).

### Bacteria

Sex differences have also been reported in bacterial infections. Analysis of 4742 randomly selected subjects, aged 12-64, from Northern Ireland shown that *Helicobacter pylori* infection was more common in males than in females (41). A retrospective seroepidemiologic survey of *Chlamydia pneumoniae* infection in patients in Beijing, China between 2008 and 2017 revealed that adult men had both a higher prevalence and higher levels of antibodies than women (42). *Klebsiella* spp. induced bacteremia was higher in males than in females in England, Wales, and Northern Ireland (43). 60% of patients hospitalized in the years 2005-2014 in USA with Lyme disease (infectious agent *Borrelia burgdorferi*) were men (44). On the other hand, reinfection with *B. burgdorferi* in individuals from Sweden that were initially diagnosed with erythema migrans and treated with antibiotics was much higher in women than in men (45). Incidence of tuberculosis that is caused by infection with *Mycobacterium tuberculosis* was described to be higher in men than in women (46), however a consistent female excess for tuberculosis at age 5-29 was observed (47). *Listeria monocytogenes* is a foodborne pathogen that is highly prevalent in pregnant woman, older adults and immunocompromised individuals. Incidence of listeriosis in the years 2008-2016 in USA was higher in males than in non-pregnant females (48). Socioeconomic factors highly influence the spread of syphilis: a sexually transmitted infection caused by *Treponema pallidum*. Sex differences in its incidence, prevalence and geographical variations have been well described. For example, the incidence of maternal syphilis is higher in low- to middle-income countries as compared to high-income countries where syphilis is more common among men who have sex with men. In Africa the spread of syphilis is also high in female sex workers (49, 50).

### Parasites

Male sex is a risk factor for visceral leishmaniasis (51). Men were more susceptible to visceral infection caused by *Leishmania donovani* (52–54) and *L. infantum* (55–58). More variability was observed in studies of sex influence on cutaneous leishmaniasis. Some epidemiological studies revealed in male patients a higher incidence of cutaneous leishmaniasis caused by *L. major* and *L. tropica* (59, 60), *L. major* only (61), and also by *L. guyanensis* (62). However, the study in Afghanistan found that females developed more lesions and scars after *L. tropica* infection (63) and other analyses reported no significant sex differences in registered cases of cutaneous leishmaniasis caused by *L. tropica* (64) and *L. major* (65). No sex bias was observed in intestinal schistosomiasis caused by *Schistosoma mansoni* in adults (66). Infection rates did not differ significantly among various age and sex groups infected with *Schistosoma haematobium* (67).

### Fungi

Prior to the AIDS epidemics, cryptococcal disease, caused by *Cryptococcus neoformans* and *Cryptococcus gattii* was rather rare. It was reported in case series 2-3 times more frequently in men as in women (68). In the AIDS era, in the years 2000-2007 were in USA hospitalized 10077 patients with cryptococcal disease, 26% were females. Males had a higher risk of a disease in both HIV-infected and uninfected cohorts. Age- and sex-adjusted death rates were almost threefold higher in males compared to females (69).

### Worms

Females were found to be more predisposed to *Ascaris lumbricoides* infection than were males (70). Human neurocysticercosis results from the infection of the central nervous system with the larval stage of the intestinal tapeworm, *Taenia solium.* In Ecuador, the number of transitional cysts in brain was found to be higher in the female than in the male patients (71).

## Various Influences on Sex Effects

Thus, differences in susceptibility and prevalence between males and females have been observed in many human infections. The extensive studies showed that some infectious diseases exhibit male (24, 25, 28–31, 41–43, 52–58, 68, 69), the other female bias (34–36, 70, 71), but there are also epidemiological studies with contradictory results; some studies showing male and the other female bias or no sex bias in the same disease (37–40, 46, 47, 59–65). These disparities may be explained by the fact that the occurrence and susceptibility to infectious diseases is influenced by many factors such as presence of pathogen reservoir, presence and properties of pathogen transmission vector in case of vector borne diseases (72), as well as immune status, sex and hormonal status, age, nutrition, microbiome and genotype of the host (72–75) and multiple environmental factors, including climate changes (76). Susceptibility to many human diseases is modified by socio-cultural determinants, behavioral/lifestyle risk factors (50), prevalence of co-morbidities (48) and co-infection with several pathogens (69, 77).

## Sex-Dependent Responses Revealed in Animal Experiments

Sex-dependent differences in response to pathogens could be more effectively analyzed in animal studies. Mouse experiments revealed important features of sex-dependent responses to infectious diseases: dose-dependence, pathogen and pathogen species-dependence, organ specificity and genetic modification.

### Dose-Dependent Sex Bias

Dose-dependent sex bias was described in responses to viruses and bacteria. The response of the strain C57BL/6 infected intranasally with the mouse adapted influenza A/PR8;H1N1 was sex-dependent when median infection dose [10^2^ or 10^3^ TCID_50_ (tissue culture infectious dose)] were used and females exhibited higher mortality than males. The effect of infections with low (10^1^ TCID_50_) or high (10^4^ or 10^5^ TCID_50_) viral inoculi was sex independent (78). Dose-dependent sex bias was observed also in the animal model of gram-negative sepsis. Wistar rats were injected intraperitoneally with bacteria *Escherichia coli* LPS in one of two doses: 1.5 or 15 mg/kg. Day after the LPS injection, serum levels of endotoxin, corticosterone, alanine aminotransferase (ALT), and aspartate aminotransferase (AST) activity in the serum and morphological changes in the lung, liver, thymus, and spleen exhibited dose-dependent sex bias. Low-dose LPS led to the serum endotoxin level increase only in males and it was combined with a more pronounced inflammatory response in the lungs (characterized by infiltration of eosinophils and neutrophils) and thymus (characterized by presence of macrophages and dead lymphocytes) and an increase and decrease in ALT and AST activity, in males and females, respectively, without any changes in corticosterone level. High-dose LPS induced systemic inflammatory response syndrome (SIRS) comprises higher blood endotoxin levels in males than in females, lower the volume fraction index of the white pulp of the spleen of males, increase of apoptotic cells in thymus and decrease of corticosteroids in males only. Sex differences of pathological changes in the lungs and liver were not revealed (79). The observed dose-dependent sex differences might be largely caused by different dynamics of induction of different signaling pathways in males and females.

### Sex Differences Depend on Species and Sub-Species of Pathogen and on Genotype of the Host

Sex bias in disease susceptibility and prevalence that is dependent on pathogen species is described in *Sex Biases in Human Infections*. Here we describe, that sex bias can depend also on pathogen sub-species. DBA/2 female mice are highly resistant and males susceptible to lesion development after infection with the parasite *L. mexicana*. On the contrary, although both female and male mice developed ulcerating lesions after infection with *L. major*, lesions healed in males, but not females (80). Sex differentially influenced also infection with *L. tropica* and *L. major* and the response was modified by genotype. Females of strains BALB/c, CcS-11, CcS-16 and CcS-20 are more susceptible than males to development of skin lesions induced by *L. tropica*, whereas no sex bias was observed in strains STS, CcS-3, CcS-5, CcS-12 and CcS-18. On the other hand, infection by *L. major* induced larger skin lesions in males of strains CcS-3, CcS-5 and CcS-18, whereas no difference between males and females was observed in strains BALB/c, STS, CcS-11, CcS-12, CcS-16 and CcS-20 (81).

### Sex Affects Pathology of Various Organs Differently and Its Influence Is Modified by the Host Genotype

Strains BALB/c and CcS-11 did not exhibit any sex influence on lesion size induced by *L. major*, but males of strain CcS-11 contained more parasites in spleens than females, and males of both strains had much higher parasite load in lymph nodes (82). Organ-dependent sex response was observed also in animal model of gram-negative sepsis (79). These phenomena might be explained by presence of different defense mechanisms in different tissues (83, 84), as well as by highly tissue-dependent sex-biases in expression of genes observed in intercross between strains C57BL/6 and C3H/HeJ (85).

## Mechanisms of Sex-Dependent Responses

The observed sex-differential responses to disease susceptibility may be explained by direct and indirect influence of sex hormones and non-hormonal sex-biasing influence of X and Y chromosomes. Sex steroid hormones (estrogen, testosterone and progesterone) influence response to infections by 1) direct effect on pathogen metabolism, growth, and expression of virulence factors. It was shown that physiological concentration of progesterone inhibited replication of *Coxiella burnetii* in JEG-3 cells (86), both testosterone and progesterone inhibited growth of *Staphylococcus aureus* (87). 2) by modification of immune response and physiology of the host. Effects on sex hormones on the host are exerted *via* sex hormone-receptor interactions. These receptors are present in cell nucleus and membrane (88) of non-immune and immune cells and tissues (88–91). Complexes of sex hormone-nuclear steroid receptor bind target DNA through hormone response elements to act as transcription factors (88). They can also bind to DNA-protein complexes and epigenetically modify cell functions (90, 91). Sex hormone-receptor complexes can exert their effects also through DNA-independent mechanisms, such as the activation of cytoplasmic signal transduction pathways (90). These interactions influence pro- and anti-inflammatory signaling pathways (92, 93). Indirect influences might include for example sex-dependent organ development (94) or influence of sex hormones on gut microbiota (95).

Non-hormonal sex-bias effects are mediated by genes localized on X and Y chromosomes (1, 96, 97). The X chromosome carries a number of immune-related genes (96), such as toll-like receptor 7 (TLR7) and interleukin-1 receptor-associated kinase 1 (IRAK1), as well as a number of immune-associated microRNAs (96). X inactivation, or silencing of one X chromosome, in women would be expected to provide dosage compensation of X-linked genes, however certain regions of the X chromosome escape inactivation (96, 98). This can lead to higher transcription levels of specific genes that are involved in sex-specific responses (96, 99). The Y chromosome also influences immune gene expression, regulation, and susceptibility to infections (97). For example, the Y chromosome mediates susceptibility to cocksackie virus independently of serum testosterone level (100). Genetic variation in chromosome Y regulates susceptibility to influenza A virus infection (101).

## Mouse Autosomal Genes That Control Sex-Biased Responses to Infections

**Figure 1:**
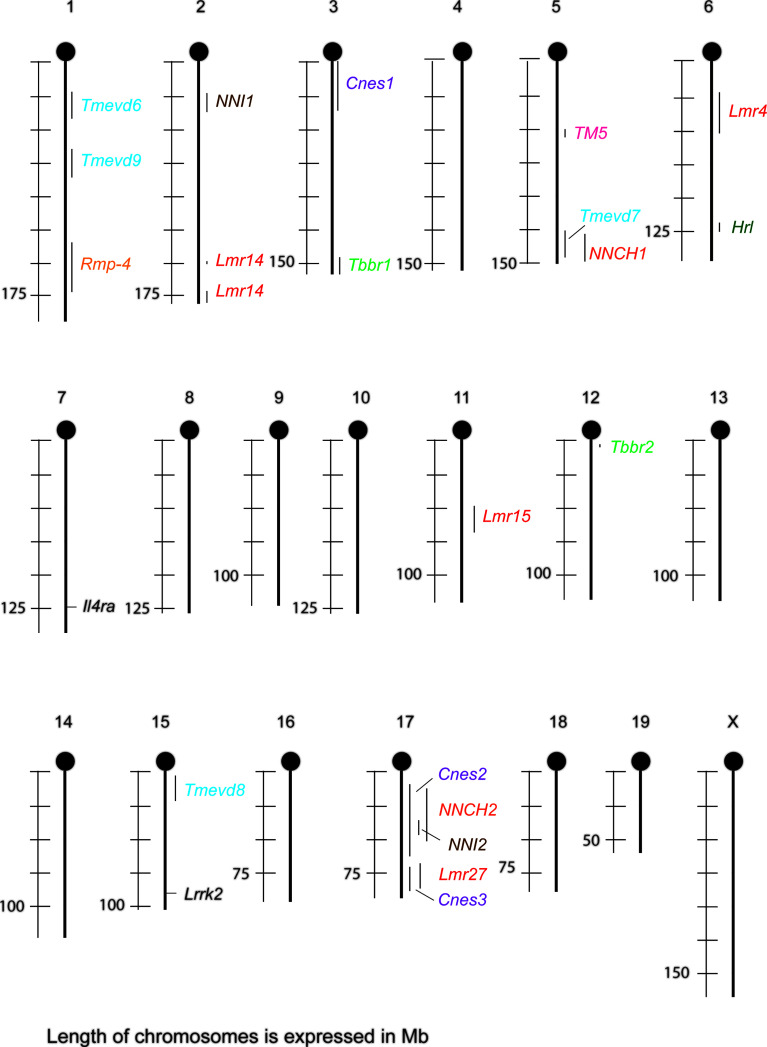
Sex-dependent loci and genes that control susceptibility to infections in mouse and their overlaps. *Cnes*, *Cryptococcus neoformans* susceptibility; *Hlr*, herpes resistance locus; *Il4ra*, interleukin 4 receptor alpha; *Lmr*, *Leishmania major* response; *Lrrk2*, leucine-rich repeat kinase-2; *NNCH*, not named *Chlamydia*; *NNI*, not named influenza; *Rmp-4*, Resistance mousepox 4; *Tbbr*, *Trypanosoma brucei brucei* response; *TM*, *Trichuris muris*; *Tmevd*, Theiler’s murine encephalomyelitis virus-induced demyelination.

Besides X- and Y-linked genes, there are also autosomal genes operating in sex-dependent manner. Sex-dependent autosomal genes modify response to viruses (102–106), bacteria (106, 107), parasites (108–111), fungi (112) and worms (113) (**Table 1**, **Figure 1**). Three models were introduced to explain gene-sex-interactions (114). 1. “Environment specific effect”: Sex dependent gene/loci affect susceptibility only in females (102, 104, 107, 111, 112), or males (102–105, 108–110, 112, 113). 2. “A main effect” model for gene by environment (= sex) interaction. A disease can affect both sexes, but is more severe in one sex compared to the other (106, 107, 111). 3. “A flip-flop” model of gene by environment interaction. Gene affects susceptibility in both sexes, but in different directions (102, 108).

**Table 1:** Autosomal genes and loci controlling sex-biased responses to infection in mouse.

Pathogen	Locus/Gene	Chromosome	Cross/Strain	Trait (phenotype) controlled	Sex effect	Reference
**Viruses**						
Theiler’s murine encephalomyelitis virus	*Tmevd6*	1	BALB/c x DBA/2J	virus-induced demyelination	opposite effects on females and males	(102)
	*Tmevd7*	5	BALB/c x DBA/2J	virus-induced demyelination	males	(102)
	*Tmevd8*	15	BALB/c x DBA/2J	virus-induced demyelination	males	(102)
	*Tmevd9*	1	BALB/c x DBA/2J	virus-induced demyelination	females	(102)
mousepox/ectromelia	*Rmp-4*	1	C57BL/6 x D2	virus titer in spleen and liver, survival	males	(103)
mouse-adapted influenzaH3N2/HK/1/68	*NNI1*	2	C57BL/6J x A/J	survival	females	(104)
	*NNI2*	17	C57BL/6J x A/J	survival	males	(104)
herpes simplex virus 1	*Hrl*	6	BALB/c x 129S6	survival	males	(105)
reovirus - T3D	*Lrrk2*- knockout	15	C57BL/6	mortality from encephalitis	females - increased mortality in knockouts	(106)
**Bacteria**						
*Chlamydia pneumoniae*	*NNCH1*	5	C57BL/6J x A/J	chlamydial burden in lungs	females	(107)
	*NNCH2*	17	C57BL/6J x A/J	chlamydial burden in lungs	stronger effects on males	(107)
*Salmonella typhimurium*	*Lrrk2*- knockout	15	C57BL/6	bacterial replication in spleen	female knockouts higher bacteria replication than WT; knockin of pG2019S mutation lower bacteria replication than WT, stronger effect on females	(106)
**Parasites**						
*Leishmania major*	*Lmr4*	6	BALB/c x CcS-9	parasite load in lymph nodes	males	(108)
	*Lmr14*	2	CcS-9 x BALB/c	eosinophil infiltration into lymph nodes	males	(109)
	*Lmr14*	2	BALB/c x CcS-9	parasite load in lymph nodes	males	(108)
	*Lmr15*	11	BALB/c x CcS-9	parasite load in lymph nodes	opposite effects on females and males	(108)
	*Lmr27*	17	BALB/c x CcS-9	parasite load in lymph nodes	males	(108)
*Leishmania mexicana*	*Il4ra* CD4+ T cell specific expression	7	BALB/c	skin lesions	non-healing phenotype in males	(110)
*Trypanosoma brucei brucei*	*Tbbr1*	3	BALB/c x CcS-11	survival	females	(111)
	*Tbbr2*	12	BALB/c x CcS-11	survival	stronger effect on females	(111)
**Fungi**						
*Cryptococcus neoformans*	*Cnes1*	3	C57BL/6J x CBA/J	lung fungal burden	females	(112)
	*Cnes2*	17	C57BL/6J x CBA/J	lung fungal burden	females	(112)
	*Cnes3*	17	C57BL/6J x CBA/J	lung fungal burden	males	(112)
**Worms**						
*Trichuris muris*	*TM5*	5	C57BL/6J x DBA/2	Serum IFN*γ*	males	(113)

The Table summarizes position on chromosome, cross used to map certain locus or mouse genetic background, disease phenotype controlled and sex effect. Cnes, Cryptococcus neoformans susceptibility; Hlr, herpes resistance locus; Il4ra, interleukin 4 receptor alpha; Lmr, Leishmania major response; Lrrk2, leucine-rich repeat kinase-2; NNCH, not named Chlamydia; NNI, not named influenza; Rmp-4, Resistance mousepox 4; Tbbr, Trypanosoma brucei brucei response; TM, Trichuris muris; Tmevd, Theiler’s murine encephalomyelitis virus-induced demyelination.

### Viruses

Theiler’s murine encephalomyelitis virus-induced demyelination (TMEVD) is an animal model for virally triggered multiple sclerosis. QTLs (quantitative trait loci) *Tmevd7* and *Tmevd8* modify susceptibility to virus-induced demyelination in males only, *Tmevd9* influences susceptibility to this disease in females and *Tmevd6* affects susceptibility in both sexes, but has an opposite effect on males and females (102). Locus *Rmp-4* (Resistance mousepox 4) modifies virus titer in spleen and liver as well as survival after infection with ectromelia virus (mousepox) (103). Two loci not named by authors *NNI1* (not named influenza 1) and *NNI2* control survival after infection with the mouse-adapted influenza H3N2/Hk/1/98. *NNI1* and *NNI2* operate in females and males, respectively (104). Susceptibility to HSV1 is in males controlled by *Hlr* (herpes resistance locus) (105). Gene *LRRK2* (leucine-rich repeat kinase-2) is a 280 KDa, multi-domain protein that has dual catalytic and kinase activity as well as number of protein-protein interaction domains. Two major inflammatory pathways have been biochemically linked to LRRK2 action: TLR pathway and NFAT pathway (115). It is associated with Parkinson’s disease, leprosy and Crohn’s disease that are disorders with an important inflammatory component. Shutinoski and co-workers tested hypothesis that *Lrrk2* plays role also in infections with paramount inflammatory responses such as reovirus and *Salmonella typhimurium* (will be discussed in the next sub-section). The increase of mortality caused by reovirus-induced encephalitis in *Lrrk2*-knockout mice in comparison with wild type animals was observed in female, but not in male mice (106).

### Bacteria

*Chlamydia pneumoniae* causes a variety of respiratory diseases. Susceptibility to this pathogen was controlled by two sex-dependent QTLs: *NNCH1* (not named *Chlamydia 1*) and *NNCH2*. Effect of *NNCH1* was observed in females, whereas *NNCH2* exerted stronger effect on males (107). Comparison of replication of *S. typhimurium in* spleens of wild type and *Lrrk2*-knockout mice shown increased replication of bacteria in spleen of female knockouts. Knockin of Parkinson’s Disease (PD)-linked p.G2019S *Lrrk2* mutation led to lower pathogen burden in spleens. The effect was stronger in females (106).

### Parasites

Sex-dependent QTLs operating in *L. major* infected mice are involved in control of pathogen load in lymph nodes (108) and infiltration of eosinophils into lymph nodes (109). *Lmr4* (*Leishmania major* response) and *Lmr27* control parasite load in lymph nodes in males, *Lmr14* influences both parasite load in and eosinophil infiltration into lymph nodes in males and *Lmr15* determines parasite load in lymph nodes in both sexes, but with opposite direction of effect (108, 109). Wild type BALB/c mice infected with *L. mexicana* develop non-healing, progressively growing skin lesions. Monitoring the course of infection with *L. mexicana* in BALB/c mice lacking expression of IL-4Rα (interleukin 4 receptor, alpha) in CD4^+^T cells revealed that these mice developed small lesions, which subsequently healed in females, but persisted in males (110). *Tbbr1* (*Trypanosoma brucei brucei* response 1) and *Tbbr2* control survival after infection with *T. b. brucei.* Effect of *Tbbr1* is visible only in females, *Tbbr2* has stronger effect on females than on males (111).

### Fungi

*Cryptococcus neoformans* is a fungal pathogen that causes pneumonia, meningitis and disseminated disease in immunocompromised host (68, 112). Fungal burden in lungs after infection with this pathogen was controlled by three sex-dependent QTLs. *Cnes1* (*Cryptococcus neoformans* susceptibility 1) and *Cnes2* operate in females, whereas effect of *Cnes3* is observed in males (112).

### Worms

*TM5* (*Trichuris muris* 5) is associated with IFNγ production in serum of males infected with parasitic nematode *T. muris* (113).

### Overlaps and Features of Sex-Dependent Loci

Some sex-dependent loci co-localize (**Figure 1**). Locus *Tmevd7* on chromosome 5 (102) overlaps with locus controlling susceptibility to *Chlamydia* (107). *Cnes2* on proximal and central part of chromosome 17 (112) co-localizes with loci modifying susceptibility to *Chlamydia* (107) and influenza (104). *Cnes3* on distal part on chromosome 17 (112) overlaps with *Lmr27* (108). This suggests the presence either of clusters of functionally related genes, or of genes that are involved in controlling the response to several infections, similarly as *Lrrk2* that controls response to reovirus and bacteria *S. typhimurium* (106).

Loci *Lmr15* (108) and *Tmevd6* (102) exhibit different effect on males and females (flip-flop model) (114) (**Table 1**). Both of them are localized on rather long chromosomal segments, thus we cannot exclude existence of two closely linked genes – one controlling susceptibility of males, the other females. However, it cannot be excluded that the opposite sex-dependent effects are controlled by one gene. Similar situation was described in humans. Polymorphism in rs2069885 (c.350 C>T) in *IL9* (5q31.1) has an opposite effect on the risk of severe respiratory syncytial virus infection in boys and girls (116), as well as on *Aspergillus fumigatus*-induced allergic lung inflammation estimated as IgE level and *IL9/IL9R* mRNA ratio in lung expectorates in males and females suffering by cystic fibrosis (117). The inflammation is stimulated by IL-9 – IL-9R on mast cells – innate lymphoid cells – Th9 pathway (117). Sex influence might be exerted by interaction of IL-9 with IL-9R. *IL9R* is located in the pseudoautosomal regions 2 (PAR2) on Xq28 and Yq12 that behave as autosomes, recombine during meiosis and PAR regions on X escape silencing (118, 119).

## Conclusions and Perspective

Sex differences in response to infections are frequent in human and form a considerable part of interpretation heterogeneity. The genetic studies in mice revealed 22 genes/QTLs influencing these differences, suggesting a genetic heterogeneity of this phenomenon. The mechanisms of effects of these sex-specific mouse genes/QTLs are unknown, but may appear as a result of sex hormone regulation of the polymorphic genes underlying these QTLs or interaction between X- or Y-chromosome-linked genes (96–99). Some of the differences between females and males might be due to sex-specific genetic architecture, characterized by profound gene-sex interactions (85, 117, 120, 121). This would mean that some genes controlling response to infections might operate differently in the two sexes. The understanding of these sex- dependent responses could facilitate personalized medicine that would take into account sexual dimorphism in susceptibility to infectious diseases, outcome of vaccination and response to treatment.

## Author Contributions 

ML and PD wrote the paper. Both authors contributed to the article and approved the submitted version.

## Funding

This work was supported by the by the Czech Science Foundation (Grant GACR 16-22346S), the Czech Academy of Sciences (RVO 68378050) and the Ministry of Health (Grant NV19-05-00457).

## Conflict of Interest

The authors declare that the research was conducted in the absence of any commercial or financial relationships that could be construed as a potential conflict of interest.

## Publisher’s Note

All claims expressed in this article are solely those of the authors and do not necessarily represent those of their affiliated organizations, or those of the publisher, the editors and the reviewers. Any product that may be evaluated in this article, or claim that may be made by its manufacturer, is not guaranteed or endorsed by the publisher.
